# Therapy outcome of day treatment for people with anorexia nervosa before and during the COVID‐19 pandemic

**DOI:** 10.1002/brb3.2604

**Published:** 2022-05-19

**Authors:** Anna Carr, Cindy Toloza, Zhuo Li, Bruno Palazzo Nazar, Hubertus Himmerich

**Affiliations:** ^1^ South London and Maudsley NHS Foundation Trust London UK; ^2^ Department of Psychological Medicine, Institute of Psychiatry, Psychology & Neuroscience King's College London London UK

**Keywords:** anorexia nervosa, day treatment, COVID‐19, pandemic, remote treatment

## Abstract

**Objective**: The current research aimed to compare clinical outcome measures of two National Eating Disorder (ED) Day Services at the Maudsley Hospital from before the COVID‐19 lockdown, when treatment was face to face, with after the lockdown when treatment moved online.

**Method**: Clinical outcome measures collected as part of the admission and discharge process were compared from the beginning and end of treatment for patients treated either via face‐to‐face or online delivery. Twenty‐nine patients’ data were analyzed (89% of them female, 11% male, 89% from White ethnic backgrounds, 11% from BAME ethnic backgrounds and a mean age of 25.99 years). Additionally, the mean change in outcome measures was also compared between the two groups (pre‐lockdown face to face and during lockdown online).

**Results**: Treatment delivered face to face led to significant improvements in body mass index (BMI) but not in Eating Disorder Examination Questionnaire (EDEQ) Global and Work and Social Adjustment Scale (WSAS) Total scores. In contrast, treatment delivered online led to significant improvements in EDEQ Global and WSAS Total scores but not in BMI. Neither one of the delivery modalities created significantly larger mean changes in any of the clinical outcome measures than the other.

**Conclusions**: Both face‐to‐face and online delivery of eating disorder day treatment show some success. Suggested improvements for using online delivery of treatment include implementing additional support opportunities, adapting the online format to improve communication and commitment and using a hybrid model of specific face‐to‐face elements with some online treatment.

## Graphical abstract

The research compares clinical outcome measures of two eating disorder day services, taken at the beginning and end of treatment, from before the COVID‐19 lockdown when treatment was face‐to‐face, with after the lockdown when treatment moved online. Face‐to‐face treatment led to significant improvements in BMI but not in EDEQ Global and WSAS Total scores, whilst online treatment led to significant improvements in EDEQ Global and WSAS Total scores but not in BMI. Online treatment improvements are suggested and a hybrid model of both face‐to‐face and online proposed.

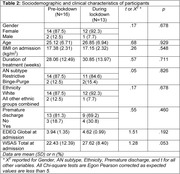

## 1 INTRODUCTION

The World Health Organization (WHO) categorized the novel coronavirus (COVID‐19) as a global pandemic on March 11, 2020. Following on from this, guidance on adjusting public health and social measures were implemented on a worldwide scale (WHO, 2020), including working from home, closure of schools and non‐essential shops, social distancing, and quarantine. Since then, studies examining the psychological impact on the general population have emerged. A review by Serafini et al. (2020) found that subjective wellbeing has decreased as a result of these restrictive measures due to psychological reactions such as pervasive anxiety, frustration and boredom, disabling loneliness, significant lifestyle changes, and previous psychiatric conditions. Similar results have been found for individuals with pre‐existing mental health conditions where there has been increased levels of social isolation, loneliness, anxiety, with reduced healthcare provided for conditions other than COVID‐19 (Mansfield et al., 2021; Rains et al., 2021).

To address the increased mental health needs of the public, some services made the transition of in‐person to online/video‐based treatments. The results thus far have been promising particularly for therapy delivery, as studies have shown a reduction in depression symptom severity, child behavior problems, and good user satisfaction (Helps & Grinney, 2021; Luo et al., 2020; McLean et al., 2021). It has been concluded that video‐based treatments are an effective way of delivering therapy due to its advantages such as being more accessible by removing physical barriers to attending face‐to‐face clinical appointments (Luo et al., 2020; Thomas et al., 2021).

Individuals with eating disorders (EDs) have been particularly impacted by the introduction of COVID‐19 measures. EDs have the highest mortality rate among psychiatric disorders with anorexia nervosa (AN) being the highest in adolescence (Arcelus et al., 2011). As a result of restrictions, inpatient treatment was provided only for severe cases of EDs, with some day hospitals and outpatient facilities becoming unavailable due to closure. Hence, the impact of the pandemic on patients with EDs overall has been detrimental to their psychological and physical wellbeing, with increased feelings of social isolation and anxiety, enhanced rumination about disordered eating, and exacerbated ED symptoms (Branley‐Bell & Talbot, 2020; Fernández‐Aranda et al., 2020; Phillipou et al., 2020). Such results highlight the urgency felt for services to adapt in order to meet the rising needs of patients with EDs.

ED units and services were also able to rapidly implement the use of technological interventions to provide online day treatment programs for ED patients which has been imperative for intensive day treatment programs for EDs. Studies have identified some positive outcomes to adapting ways of working with ED patients such as greater capacity and accessibility of treatment, willingness from patients to engage in them, and improved patient–clinician communication (Plumley et al., 2021; Shaw et al., 2021; Weissman et al., 2020). However, it is important to acknowledge other factors of online treatment that may exacerbate ED symptoms such as loss of treatment support, detached online connection due to practical difficulties, reduced rapport/therapeutic alliance, and patients being better able to hide/mask their symptoms (Couturier et al., 2021; Fernández‐Aranda et al., 2020; Shaw et al., 2021; Vuillier et al., 2021).

Therefore, the current report aims to investigate the impact of the move to online from face‐to‐face treatment during the COVID‐19 lockdown using clinical outcome measures in an ED day service.

## 2 METHOD

### 2.1 Aims and objectives

The current study aims to investigate the move from in person to online delivery of ED treatment during the COVID‐19 lockdown. Our primary objective was to compare clinical outcome measures taken at the beginning and end of treatment at the 2‐day service programs for patients treated pre‐lockdown (face to face) and during lockdown (online). Our secondary aim was to compare the change in clinical outcome measures between the two groups (pre‐lockdown and during lockdown), in order to evaluate how the type of treatment (face‐to‐face or online) impacts the change in clinical outcomes.

### 2.2 Participants

All participants for the study were treated in the Step Up or Day Care Eating Disorder Day Services either between February 2, 2018 and January 3, 2020, forming the pre‐COVID lockdown group, or from September 17, 2019 to May 19, 2021 forming the during COVID lockdown group. Where patients’ treatment started before the COVID outbreak but continued into restrictions, they were classified into pre‐ or during COVID groups based on how the majority of their treatment was delivered. Throughout the “during lockdown” timeframe, there were generally quite high levels of COVID‐19 restrictions in place, excluding some short pockets of time where some restrictions were lifted. All patients included received treatment before the end of the government's “roadmap” out of lockdown and before restrictions began being permanently lifted. Considering the length of time spent in treatment on average, all patients receiving online treatment will have had a substantial amount of their treatment during periods of high COVID‐19 restrictions. Participant ages ranged from 18 to 45. All participants had a diagnosis of AN restrictive type or AN binge‐purge type, according to the 5th edition of the Diagnostic and Statistical Manual of Mental Disorders (DSM‐5; American Psychiatric Association, 2013). Other diagnoses (Bulimia Nervosa and Eating Disorder Not Otherwise Specified) were excluded due to small sample sizes. Anyone who “completed” treatment (received treatment and was discharged) in the service during the times specified, who completed admission and discharge outcome measures and who had a diagnosis of AN met the participant criteria and their data were used.

### 2.3 Interventions

The intervention discussed in the current study is an intensive day service provided by National Eating Disorder Services at the Maudsley Hospital, which is split into two parallel streams called day care (DC) and step up (SU). Both services provide intensive day patient treatment for individuals with EDs who require a supported step down in care from inpatient services or a more intensive intervention than an outpatient service; however, they run slightly differently to meet the needs of the patient group. Both services transitioned to an online‐based treatment on March 16, 2020 and were delivered using Microsoft Teams. See Table 1 for a summary of treatment provision in each service.

**TABLE 1:** Summary of treatment provision in each service (SU and DC), and whether it is offered to all patients (“all”), on an individual basis (“individual”) or not at all (“none”)

	In person treatment		Virtual treatment	
Intervention	SU	DC	SU	DC
Meal support	All	All	All	All
Post meal support	All	All	All	None
Psychoeducational groups	All	All	All	All
Creative groups	All	All	All	All
Practical groups (e.g., cooking and buying snacks out)	All	All	None	None
Goal setting group	All	All	All	All
Trouble shooting group	None	None	All	None
1:1 Check in calls	N/A	N/A	All	All
Group mentalization‐based therapy	None	All	None	All
Individual Acceptance Commitment Therapy (if warranted)	None	None	None	Individual
Individual cognitive remediation therapy (if warranted)	None	None	Individual	None
Other individual psychological therapy	None	All	Individual	All

Abbreviations: DU, day care; SU, step up.

The DC stream continued to offer groups similar to its in‐person program following the move online, including psychoeducational, creative, goal setting, and therapeutic groups. Meal support continued, however, was adapted to become an optional social eating group (whereby patients could join a group video call whilst eating). Individual outpatient therapy was continued for some patients; however, due to the pandemic, some patients were put on a waiting list for therapy. Those waiting were offered short‐term “pre‐therapy treatment” based on Acceptance Commitment Therapy (ACT). Weekly individual check in calls were also introduced for the online program. The boundaries in DC remained the same, whereby patients were required to gain a minimum of 1.2 kg a month and were expected to attend groups on treatment days. If these boundaries were not met, patients were given a reflective week out from the program.

SU also remained similar to its in‐person program, with groups similar to DC plus an additional trouble shooting group every morning and individual check in calls. The move online also increased the resource for patients to be offered individual cognitive remediation therapy (CRT) or, if a need was identified, individual psychological therapy. Patients in the SU stream were expected at the very least not to lose weight and encouraged to be committed to weight restoration, with boundaries set more individually. If the program expectations were consistently not being met, the patient would be invited to a review to think about whether the program is meeting their current needs or whether a different care pathway should be considered.

As a result of the online delivery of both streams, some practical groups were lost such as cooking or buying snacks out. There was also less space for patients to socialize outside of the intensive program. After the lockdown, physical health checks were no longer able to be carried out in person with the service and so patients saw their own GPs for this when necessary and were required to self‐report their own weight. Both teams continued to discuss and plan the best care for individual patients in management rounds and/or group clinical supervision.

### 2.4 Measures

All patients’ BMIs are measured upon admission and discharge to and from the services. Demographic information is also collected routinely upon admission to and discharge from the service and can be seen in Table 2. All other clinical measures were taken from questionnaire packs completed by patients upon admission and discharge from the services. Each program gives slightly different routine questionnaires at admission/discharge; therefore, the current study selected the overlapping measures that both programs used routinely. The following measures were included.

**TABLE 2:** Sociodemographic and clinical characteristics of participants

	Pre‐lockdown (*n* = 16)	During lockdown (*n* = 13)	*t* or χ^2a^	*p*
Gender			.17	.678
Female	14 (87.5)	12 (92.3)		
Male	2 (12.5)	1 (7.7)		
Age	25.12 (6.71)	26.85 (6.94)	.68	.929
BMI on admission (kg/m^2^)	17.38 (2.31)	17.15 (2.32)	.26	.548
Duration of treatment (weeks)	28.06 (12.49)	30.85 (13.97)	.57	.711
AN subtype			.05	.826
Restrictive	14 (87.5)	11 (84.6)		
Binge‐Purge	2 (12.5)	2(15.4)		
Ethnicity			.17	.678
White	14 (87.5)	12 (92.3)		
All other ethnic groups combined	2 (12.5)	1 (7.7)		
Premature discharge			.55	.460
No	13 (81.3)	9 (69.2)		
Yes	3 (18.7)	4 (30.8)		
EDEQ Global at admission	3.94 (1.35)	4.62 (0.99)	1.51	.192
WSAS Total at admission	22.43 (12.39)	27.62 (8.40)	1.28	.053

*Note*: Data are mean (SD) or *n* (%).

Abbreviations: BMI, body mass index; EDEQ, Eating Disorder Examination Questionnaire; WSAS, Work and Social Adjustment Scale.

^a^χ^2^ reported for Gender, AN subtype, Ethnicity, Premature discharge, and *t* for all other variables. All Chi‐square tests are Egon Pearson corrected as expected values are less than 5.

#### 2.4.1 *Eating Disorder Examination‐Questionnaire* (Fairburn & Beglin, 1994)

The Eating Disorder Examination‐Questionnaire (EDE‐Q) is a 36‐item self‐report questionnaire that provides an assessment of the range and severity of eating disorder psychopathology. It is scored on four subscales: restraint, eating concern, weight concern, and shape concern, the mean of which gives a Global EDE‐Q score. The Global score has been shown to have a test re‐test reliability of.92 (Rose et al., 2013) and will be the measure reported in the current study.

#### 2.4.2 Work and Social Adjustment Scale

The Work and Social Adjustment Scale (WSAS) is a 5‐item self‐report questionnaire measuring impairment to work and social functioning. Higher scores indicate higher levels of impairment with a score of 20+, indicating impairment that is moderately severe or worse. Scores between 10 and 20 are associated with significant, but less severe impairment and scores below 10 are deemed subclinical (Mundt et al., 2002). It is specified to patients that ratings should reflect impairment secondary to their EDs.

### 2.5 Statistical analysis

SPSS statistics was used to collaborate data, calculate descriptive statistics, and run the tests for statistical analyses.

Baseline characteristics were compared between patient groups using Student's *t*‐test and Chi‐square test. Egon Pearson correction was applied where expected values were less than 5, as recommended by Campbell (2007). As primary analysis, paired‐samples *t*‐tests were conducted to compare patients’ clinical outcome measures (BMI, Global EDEQ score, and WSAS score) before and after treatment. Treatment was either in a face‐to‐face day service before the COVID‐19 lockdown, or in a virtual day service during the COVID‐19 lockdown. For the secondary analysis, the change in BMI was compared between the pre‐COVID and during COVID groups using an ANCOVA with the pre‐specified covariates of age, sex, and BMI at admission. Similarly, changes in EDEQ Global and WSAS Total scores were compared between groups using ANCOVA with the covariates age, sex, BMI at admission, and the value of the respective variable at the time of admission.

## 3 RESULTS

From an initial sample of 55, 29 patients were included in the study. This was due to some patients not completing outcomes measures on their discharge or still being in treatment at the time of the study. See Table 2 for participants’ sociodemographic and clinical characteristics. The groups did not differ in regard to gender, age, admission BMI, treatment duration, AN subtype, ethnicity, rate of premature discharge, admission EDEQ Global score, or admission WSAS Total score.

### 3.1 Primary analysis

A paired‐samples *t*‐test was conducted to compare BMI, EDEQ Global, and WSAS Total scores at the beginning and end of treatment in both groups (pre‐COVID lockdown and during COVID lockdown). See Table 3 for descriptive statistics.

**TABLE 3:** Descriptive statistics for clinical outcomes before treatment (on admission), after treatment (on discharge), and overall change in patients treated before and during the COVID‐19 lockdown

	Pre‐COVID lockdown (face‐to‐face) group			During COVID (online) group		
	Admission	Discharge	Change	Admission	Discharge	Change
BMI	17.38 (2.31)	18.44 (2.76)	1.07 (1.64)	17.15 (2.32)	17.64 (2.86)	.396 (1.47)
EDEQ Global	3.94 (1.35)	3.36 (1.67)	−.404 (1.96)	4.62 (0.99)	3.66 (1.20)	−.769 (1.22)
WSAS Total	22.43 (12.39)	19.44 (10.36)	−1.31 (8.51)	27.62 (8.40)	19.38 (9.54)	−6.36 (10.71)

*Note*: Data are mean (SD).

Abbreviations: BMI, body mass index; EDEQ, Eating Disorder Examination Questionnaire; WSAS, Work and Social Adjustment Scale.

### 3.2 Pre‐COVID lockdown (face to face) group

There was a significant difference in BMI measures before compared to after treatment; *t*(15) = −2.59, *p* = .021.

No significant difference was found in EDEQ Global scores before compared to after treatment; *t*(15) = 1.92, *p* = .074) or in WSAS Total scores before compared to after treatment; *t*(15) = 1.41, *p* = .180).

### 3.3 During COVID lockdown (online) group

There was no significant difference in BMI measures before compared to after treatment; *t*(12) = −1.19, *p* = .258.

However, significant differences were found in EDEQ Global scores before compared to after treatment; *t*(12) = 3.36, *p* = .006, and in WSAS Total scores before compared to after treatment; *t*(12) = 3.31, *p* = .006.

### 3.4 Secondary analysis

Secondary analysis was conducted to compare the mean change in each outcome measure (BMI, EDEQ Global, and WSAS Total) from the beginning to the end of treatment, between the pre‐COVID group compared to the during COVID group. This was done using a linear model adjusted for age, sex, and baseline value. The assumption of homogeneity of variances was tested and satisfied for all outcome measures, BMI, EDEQ Global, and WSAS Total, using a Levene's Test for Equality of Variances, *F*(28) = .04, *p* = .843, *F*(28) = 1.71, *p* = .202, and *F*(28) = .26, *p* = .615, respectively. See Table 3 for descriptive statistics.

### 3.5 Change in BMI

There was no significant difference in the mean change of BMI scores in the pre‐COVID group compared to the during COVID group, *F*(1, 24) = .53, *p* = .475.

### 3.6 Change in EDEQ Global Scores

The difference in the mean change of EDEQ Global scores in the pre‐COVID group compared to the during COVID group was not significant (*F*(1, 23) = .54, *p* = .469).

### 3.7 Change in WSAS Total Scores

There was no significant difference in the mean change of WSAS Total scores in the pre‐COVID group compared to the during COVID group (*F*(1, 23) = 2.97, *p* = .098).

## 4 DISCUSSION

The current study compares clinical outcome measures for patients treated in ED day services before the COVID‐19 lockdown (when the service was face to face) with the outcome measures for patients treated during the lockdown (when the services switched to being online). The study aimed to investigate whether ED day treatment differed in success regarding weight restoration and other measures of clinical improvement when it was delivered in a face to face compared to an online modality. A secondary aim was to compare the change in outcome measures between the two groups (pre‐lockdown and during lockdown) and to evaluate how the type of treatment (face to face or online) might impact the change in clinical outcomes.

For those treated face to face, BMI measures significantly increased from the beginning of treatment compared to the end, though EDEQ and WSAS scores did not. In contrast, the group treated online showed no significant differences in BMI at the beginning compared to the end of treatment; however, EDEQ and WSAS scores significantly improved. These results suggest the potential for some areas that are important for recovery to be treated using an online delivery modality. It is well cited that people with anorexia ruminate more about food and their bodies (Seidel et al., 2018) and generally accepted that in addition to physical/behavioral measures, psychological symptoms are also important to address in recovery (Bardone‐Cone et al., 2018). Research suggests a general trend for weight restoration preceding psychological recovery from EDs (Khalsa et al., 2017), which might suggest why in the face‐to‐face treatment group, patients showed significant increases in weight but not in EDEQ and WSAS scores. However, in the present study, overall eating, weight, and shape concern (as measured by the EDEQ) significantly decreased following the online treatment, indicating possible benefits of an online intervention which includes improving psychological wellbeing in EDs. Still, it should be noted that weight restoration is a crucial part of ED recovery and a major factor relating to shorter hospital admissions, successful transference out of hospital and into day services, and lower readmission rates (Gjoertz et al., 2020).

The study also found significant improvements in WSAS scores following online treatment. This is important given the role that social wellbeing plays in general wellbeing and mental health as a whole (De Vos et al., 2018), however, a surprising contrast to existing research highlighting the negative impact of the lockdown on peoples’ lifestyle and levels of isolation and loneliness (Serafini et al., 2020). Given the unprecedented effects the COVID‐19 pandemic has had on peoples’ wellbeing and functioning, it is important to interpret the current results with caution and consider where there may be alternative explanations for results. For example, one study that surveyed 159 patients with AN found that some positive consequences of the COVID‐19 pandemic were reported, including having more flexibility and learning to tolerate some uncertainty (Schlegl et al., 2020). Considering this, one cannot rule out the potential for positive effects of the lockdown (being more flexible and tolerating more uncertainty) to improve some AN patients’ social functioning that may be reflected in WSAS scores. Additionally, given that interpersonal issues serve as a maintaining factor for EDs (Harrison et al., 2014), improving social engagement and adjustment is also an important area to address during ED recovery. The significant improvement seen in WSAS scores following online treatment in the current study suggests that online treatment can potentially support patients with their work and social functioning.

Comparing the mean change in BMI, EDEQ Global, and WSAS Total scores at the beginning and end of treatment between the groups while controlling for potential covariates showed that there were no significant differences in the mean change of any of the outcome measures between the pre‐COVID compared to the during COVID group. This indicates that neither modality of treatment delivery led to significantly larger changes in clinical outcome measures over treatment duration than the other, despite some significant changes from pre‐ to post‐treatment measures within groups. In other words, receiving treatment did significantly impact the clinical outcomes from pre‐ to post‐treatment within both groups (online and in person treatment); however, overall, the type of treatment (face to face vs. online) did not have a significant impact on change in clinical outcome measures.

Previous studies evaluating evidence‐based treatments being delivered online, such as cognitive behavioral therapy (CBT) or family‐based therapy (FBT), have consistently shown the efficacy, improved therapeutic alliance, and patient satisfaction of treatments being delivered online to be comparable to face‐to‐face delivery (Helps & Grinney, 2021; Luo et al., 2020; McLean et al., 2021; Norwood et al., 2018; Thomas et al., 2021). Evaluations of online treatment for EDs specifically reveal more mixed results. While some findings suggest positive outcomes for online treatment for EDs, improved access to them, willingness from patients to engage in them and improved patient–clinician communication (Shaw et al., 2021; Weissman et al., 2020), others have highlighted concerns around rapport/therapeutic alliance, reduced access to services, heightened anxiety appearing on video, lack of accountability, and being better able to hide or mask ED symptoms/behaviors (Couturier et al., 2021; Fernández‐Aranda et al., 2020; Shaw et al., 2021). Being at home due to the pandemic also allowed for more opportunity to engage in ED behaviors such as restricting food intake, binging, and purging or over‐exercising (Vuillier et al., 2021). Considering this, significant changes to BMI may not have been observed in the patients treated during the lockdown when the service moved to online due to a lack of accountability to the actions necessary to see weight gain. For example, when being treated in an in‐person service, patients are generally held more accountable to completing meals, being weighed, not exercising during the day, and so forth, compared to receiving treatment online, where crucial parts of the treatment program such as supported meals are not feasible. Additionally, while at home patients were asked to self‐report their weight. This constant awareness of their own weight, without the support and reassurance of a clinician, may serve to increase anxiety and overconcern with weight (Froreich et al., 2020), therefore negatively impacting the ability to restore weight.

The current results offer suggested advantages and disadvantages to online versus face‐to‐face treatment. It is possible that the move to an online format of treatment delivery is associated with a positive impact on EDEQ outcomes due to less competitiveness between patients and less opportunity to pick up or mimic harmful pro‐ED ideas and behaviors. Research by Feldhege et al. (2021) supports this idea by finding attitude shifts in an online ED community since the beginning of the COVID‐19 pandemic, from the promotion of pro‐ED behaviors and moving towards a more pro‐recovery orientation. On the other hand, as discussed above, a face‐to‐face program provides more accountability for example through supervised meals, which is valuable for weight restoration. Given the process of weight gain generally being a challenging and distressing experience for people with EDs (Matthews et al., 2019; Williams et al., 2021), it is suggested that in the face‐to‐face treatment group, where significant changes to BMI were seen, the distress from the weight gain may have impacted on other indications of recovery, for example, leaving little energy and mental capacity for patients to work on addressing mood, relationships, social and home life, and so forth. Importantly, the additional impact of COVID‐19 itself must also be considered here, with reported increases in worry, loneliness, restlessness, and general sadness in some individuals with AN during the COVID‐19 lockdown (Schlegl et al., 2020), which would be likely to influence ED symptoms as well as occupational and social functioning which could be reflected in EDEQ and WSAS scores.

The current study is early exploratory research into the impact of the COVID‐19 lockdown and move to online ED day services. It is limited by the small sample size and would benefit from further examination with larger sample sizes and more controlled conditions. Sample size was limited, as only patients who completed outcome measures upon ending treatment could be included; however, to maximize sample size and reduce bias where possible, all those who were admitted to the service for treatment and completed measures at the end of treatment were included, even in cases where treatment was terminated early. Another limitation that should be noted is the impact of premature discharge from day service treatment. Patients may be discharged early from the services due to disengagement, not meeting therapeutic boundaries/expectations or requiring more intensive treatment such as inpatient care. As demonstrated in Table 2, more patients were discharged early from services after moving online (during the lockdown) compared to the face‐to‐face services. This may impact the validity of the clinical measures taken at the end of treatment for these individuals due to their day service treatment being “cut short” relative to the typical duration of the programs. Additionally, the programs often took an individualized approach to care planning and treatment; therefore, patients will have received slightly different types and/or frequency of intervention throughout their treatment duration. While the study was not able to control for this variable, continuing to offer patients their individualized recommendations was important to ensure the programs were meeting ethical standards and not limiting opportunity or suggesting unnecessary additional treatment. It is acknowledged that the variation in what exact interventions were offered could have been reflected in the findings with greater or lesser improvements indicated by the measures. However, these were not controlled for in the current study which aimed to examine the outcome of the program as a whole. Additionally, it should be noted that patients may have had comorbid diagnoses, such as depression or anxiety, alongside their EDs that could have influenced findings; however, controlling for these was outside the scope of this smaller scale exploratory study. Future studies would benefit from controlling for these possible confounding variables, ideally conducting a randomized control trial (RCT) to compare the online versus in‐person programs, or indeed a three‐armed RCT to evaluate online, face‐to‐face and blended treatment options (a mixture of face to face and online).

The current findings are a promising start to further adapting and developing online treatment services for EDs. The present study indicates the potential for online treatment to be, at least in part, a successful mode for delivering ED treatment and endorses suggestions in line with other recent findings to continue improving online treatment. For example, pairing online treatment with specific face‐to‐face interventions to deliver a blended treatment approach may be beneficial and have the potential to override some of the limitations that one approach may have. This may include new patients initially meeting the care team in person to build rapport before starting the program, coming in to get weighed in order to provide accountability and reduce the anxiety around doing it on their own, and providing meal, cooking or shopping support which are not feasible to deliver online. Furthermore, the current study shows that online treatment alone was not sufficient facilitate significant changes in BMI. As discussed, weight restoration is a crucial part of recovery from an ED (Gjoertz et al., 2020); however, research indicates that weight gain alone is not sufficient to address other psychological symptoms around body dissatisfaction such as weight and shape concern (Accurso et al., 2014; Fennig et al., 2017). Considering the current study's results, it could be suggested that a treatment option combining some online and some face‐to‐face treatment has the potential to support both weight restoration alongside an improvement to psychological symptoms.

Other suggestions to improve online treatment include supplementing what the treatment offers with additional support opportunities such as extra groups or setting up open social forums and adapting the online format to improve communication and commitment. This could include ensuring groups have a clear structure and protocol, providing materials in advance or providing a summary of a group or session afterwards (Brothwood et al., 2021; Waller et al., 2020), allowing for preparation and continued self‐help. Additionally, given the importance of weight restoration in recovery (Gjoertz et al., 2020), one suggestion that may merit further exploring is the possibility for patients of very low weight to be treated in a face‐to‐face service until some progress has been made with weight restoration, after which recovery could continue with the support of online treatment.

## 5 CONCLUSION

Our data suggested that face‐to‐face ED day treatment results in higher rates of weight restoration in comparison to online ED day treatment, which showed greater improvements to psychological outcomes. Future studies with a larger sample size and that aim to investigate other potential confounding variables would be beneficial to explore the impact of these outcomes further.

## CONFLICT OF INTEREST

The authors declare that the research was conducted in the absence of any commercial or financial relationships that could be construed as a potential conflict of interest.

### PEER REVIEW

The peer review history for this article is available at https://publons.com/publon/10.1002/brb3.2604.

## Data Availability

The data presented in this study cannot be made freely and publicly available because the study participants did not consent to it. However, part of the data or results of further data evaluations may be shared at the discretion of the corresponding author in accordance with the applicable guidelines.
